# Porous polymer coatings on metal microneedles for enhanced drug delivery

**DOI:** 10.1098/rsos.171609

**Published:** 2018-04-18

**Authors:** Asad Ullah, Chul Min Kim, Gyu Man Kim

**Affiliations:** School of Mechanical Engineering, Kyungpook National University, 80 Deahak-ro, Buk-gu, Daegu 41566, Korea

**Keywords:** porous polymer coating, metal microneedle, PLGA, *in vitro* drug delivery, calcein, lidocaine

## Abstract

We present a simple method to coat microneedles (MNs) uniformly with a porous polymer (PLGA) that can deliver drugs at high rates. Stainless steel (SS) MNs of high mechanical strength were coated with a thin porous polymer layer to enhance their delivery rates. Additionally, to improve the interfacial adhesion between the polymer and MNs, the MN surface was modified by plasma treatment followed by dip coating with polyethyleneimine, a polymer with repeating amine units. The average failure load (the minimum force sufficient for detaching the polymer layer from the surface of SS) recorded for the modified surface coating was 25 N, whereas it was 2.2 N for the non-modified surface. Calcein dye was successfully delivered into porcine skin to a depth of 750 µm by the porous polymer-coated MNs, demonstrating that the developed MNs can pierce skin easily without deformation of MNs; additional skin penetration tests confirmed this finding. For visual comparison, rhodamine B dye was delivered using porous-coated and non-coated MNs in gelatin gel which showed that delivery with porous-coated MNs penetrate deeper when compared with non-coated MNs. Finally, lidocaine and rhodamine B dye were delivered in phosphate-buffered saline (PBS) medium by porous polymer-coated and non-coated MNs. For rhodamine B, drug delivery with the porous-coated MNs was five times higher than that with the non-coated MNs, whereas 25 times more lidocaine was delivered by the porous-coated MNs compared with the non-coated MNs.

## 1. Introduction

Microneedles (MNs) are an attractive alternative for hypodermic and subcutaneous needle applications that have recently gained attention [1,2]. Depending on the application, needles must have suitable length to avoid nerve contact, and smooth surface, sharp tip and high mechanical strength to penetrate skin without cracking, breaking or bending; which ensures patient compliance. Moreover, needles must also create sufficient pathways for the delivery of small drugs, macromolecules and nanoparticles, as well as to sample interstitial fluids [3,4]. To achieve these objectives, the minimally invasive drug delivery system composed of an MN array of sub-millimetre needles has been studied and developed to overcome some of the limitations usually associated with hypodermic needle usage. Generally, four types of MNs are used in a transdermal drug delivery system (TDDS) [5]: solid, coated, dissolvable and hollow. Solid MNs are very short physical needles used to puncture the skin and open micro-pathways painlessly in the skin through which a drug formulation can be introduced [6–8]. Once the skin is punctured, the drug permeates through the stratum corneum and then passes through the deeper epidermis and dermis. When the drug reaches the dermal layer, it is actively absorbed by the dermal microcirculation system [9,10]. Very short lifespans of such micro-passages are the main limitation of TDDSs using solid MNs. Another way of using solid MNs is the ‘coat and poke' method, which involves inserting drug-coated solid MNs into the skin [11,12]. Although drug delivery from coated MNs has been shown to be especially useful for high-molecular-weight molecules, this approach is rather limited due to the small dimensions of the MNs shaft and tip [13]. An array of 36 MNs was used to coat 9 µl of drug formulation and only 40% of the loaded drug was delivered. Surface texturing on the needle surface via electromechanical machining methods could be employed to increase the needle's surface area [14–18]; however, only marginal increases in the surface area have been achieved. Specifically, Ryu [14] achieved approximately 200 nm² size craters on the surface area by electromechanical machining methods [14]. The ‘poke and release' method involves the use of dissolving and hydrogel-forming MNs. These MNs are produced from materials that act as drug storage units, retaining the drug formulations until the drug dissolves (in case of dissolving MNs) or swells (in case of hydrogel MNs) [19–23]. However, this method can only be used for delivering solid drugs and it has a low delivery rate. Finally, hollow MNs have a tubular bore inside the MN body. Drug delivery by hollow MNs involves the ‘poke and flow' method whereby the MN pierces the skin to allow the drug formulation to transfer through the MN bore in a manner similar to that employed by hypodermic needles [24]. In this case, both passive diffusion and active delivery may occur. The main disadvantage of hollow MNs is the high cost of the manufacturing technology required to ensure maximum precision.

In another approach, porous MNs have a large number of randomly distributed pores that provide passages for drug delivery as well as interstitial fluid sampling. They are attractive because of their fast interstitial fluid sampling by capillary action. However, polymer porous MNs are fragile and unable to penetrate skin properly due to insufficient mechanical strength [25,26]. To address this issue, porous polymer MNs are advantageous because of their biocompatibility, structural stability and easy fabrication process. Although poly (lactic acid) microparticles have been used to fabricate porous polymer MNs, the resultant MNs were too delicate to puncture skin [27,28]. Using polymerization, in the presence of porogen, stronger MNs can be fabricated; however, the MNs produced thus have inconsistent geometry and hence are not suitable for further applications [29]. Recently, poly (glycidyl methacrylate) porous MNs were fabricated in the presence of a poly (ethylene glycol) solution by photopolymerization. Even though the synthesized MNs were found to possess good geometries and mechanical strength with 39% porosity, further increases of the porosity caused the MNs to have blunter tips, which decreases their rate of delivery [30].

**Figure 1.**
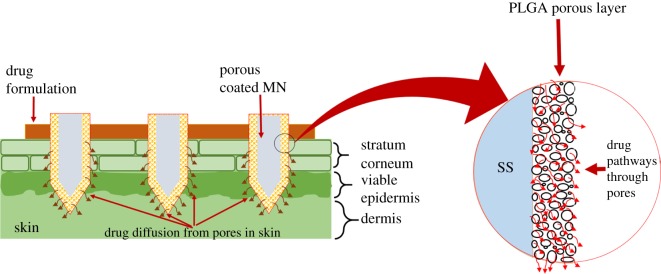
Schematic representation of the drug formulation diffusion through porous MNs in skin.

This study combines the ‘coat and poke' and ‘poke and flow’ methods to maximize their advantages while minimizing their limitations. Specifically, solid MNs were coated with a porous poly (lactic-co-glycolic acid) (PLGA) coating composed of highly interconnected pores, which allow liquid drugs to be easily transported. The pores provide an efficient pathway for the drug in a manner similar to that of hollow MNs. Additionally, because the MNs possess high mechanical strength, they are able to penetrate skin without cracking, breaking or bending. A schematic representation of the proposed drug delivery system is shown in figure 1. Notably, the polymer coating is easy to prepare and requires no particular expertise or technologies, and simply dipping the MNs in the polymer solution coated the solid MNs. In order to enhance adhesion between the polymer and MNs, the latter was exposed to plasma for 10 min and coated with polyethyleneimine (PEI) prior to application of the PLGA porous coating.

## 2. Material and methods

### 2.1. Materials

The following materials for this study were purchased from Sigma-Aldrich: poly (lactic-co-glycolic acid) (PLGA, lactide : glycolide = 65 : 35, MW: 40 000–75 000), poly (vinyl alcohol) (MW: 31 000–50 000, 98–99% hydrolysed), gelatin (from porcine skin), dichloromethane (DCM, anhydrous, ≥99.8%, *ρ*: 1.325 g ml^−1^, MW: 84.93 g mol^−1^, mp: –97°C), Sylgard 184 silicone elastomer base and agent, stainless steel wire (SS), poly (methyl methacrylate) (PMMA) also known as acrylic plastic, PEI (analytical standard, 50% (w/v) in water), rhodamine B dye, calcein dye, lidocaine (MW: 234.34 Da) and phosphate-buffered saline (PBS).

### 2.2. Coating solution preparation and porous poly (lactic-co-glycolic acid) microneedle fabrication

Two types of MNs were coated. To coat the first type of MNs (type A, obtained from a local company, ‘UBioMed’), a 25 mm square plate of PMMA was machined to a height of 3 mm and a jig was used to fashion holes for nine MNs. The height and diameter of the MNs on the array were 0.600 and 0.120 mm, respectively. An array of 3 × 3 MNs with intervals of 2 mm was prepared by inserting MNs into an acrylic plate. Fabrication of the second type of MNs (type B) used 4 mm long MNs that were cut from a SS wire with a 0.3 mm diameter. These MNs were then inserted in a polydimethylsiloxane jig. Further details about type A MNs can be found in supporting data (electronic supplementary material, figure S1). Type B MNs were used only for visual comparison of Rhodamine B dye delivery in the gelatin gel, whereas in all other experiments type A was used.

The polymer coating solution was prepared with 2% (w/w) PLGA in DCM. A 15% (w/w) aqueous gelatin solution was also prepared in a 1% polyvinyl alcohol solution. These two solutions were then mixed and homogenized at 20 000 r.p.m. for 3 min to prepare the coating solution emulsion.

The MNs were cleaned using acetone, deionized (DI) water and ethanol. The MNs were then dried prior to the plasma treatment. After drying, the MNs were placed in a plasma chamber and subjected to the discharge of oxygen plasma for 10 min. The plasma generator operated at a frequency of 13.56 MHz (RF-GEN, IDT Eng. Co., Korea), and the specifications of the plasma discharge were as follows: plasma power (260 W), time (10 min) and oxygen pressure (0.2 mTorr). After the plasma treatment, the samples were removed from the plasma chamber and submerged in a solution of 2% PEI (w/w) in water and left for 15 min. Next, the samples were submerged in DI water for 1 min in triplicate and then immersed in the PLGA and gelatin emulsion (i.e. the coating solution) for 20 min to coat the porous polymer layer on the MNs.

**Figure 2.**
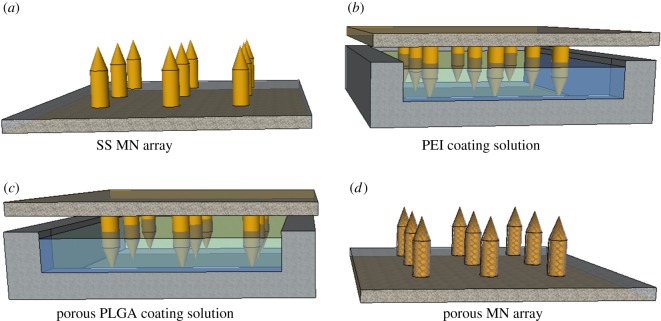
Schematic representation of the PLGA porous-coating process.

Subsequently, the samples were kept at 25°C for 2 h to evaporate the solvent completely. They were then placed in a warm water bath at 37°C for 3 h to remove the gelatin and reveal the porous polymer layer on the MNs. Figure 2 shows schematic images of these processes.

### 2.3. Peel-off tests

Peel-off tests were performed to visualize the polymer coating stability on the MN surfaces at room temperature using a universal testing machine (UTM, Instron, model 4465, USA). Two samples were prepared for these tests, which differed only in whether or not surface modification was performed. Regardless of surface modification, the samples consisted of a 0.380 mm thick PLGA layer coated onto a 15 mm wide and 100 mm long rectangular SS plate with a height of 2 mm. Each sample of the PLGA-coated SS plates with and without surface modification was fixed onto motor-driven jaws that progressively moved in one direction. The operating specifications were as follows: track length (6 mm), scratch speed (0.1 mm min^−1^), width of probe (15 mm), thickness of probe (0.3800 mm) and applied load range (0–30 N). The tangential friction force was measured during the experiments, and each test was performed in triplicate. The average values were used for the analysis.

### 2.4. Skin penetration capability of porous polymer-coated microneedles

To study the mechanical strength and insertion capability of the porous polymer layer on the MNs, an array of porous polymer-coated MNs (3 × 3) was prepared and manually inserted into the skin for 1 min and then removed. The details about skin used are given in §2.5. Scanning electron microscopy (SEM) was used to visualize the porous coatings on the MNs before and after insertion.

### 2.5. Calcein dye delivery in porcine skin

Full thickness fresh porcine skin pieces were sourced from a local abattoir and immediately stored at −20°C. Before the experiments, skin pieces were thawed for 1 h at room temperature. The hair was carefully cut using hair clippers and the skin surfaces were washed carefully with DI water and dried completely before use. Skin pieces were placed on a fixed platform and pinned down to maintain slight tension across the surface.

Both porous-coated and non-coated MNs were used to deliver calcein through the skin. Briefly, porous polymer-coated and non-coated MNs were dipped in 5% calcein aqueous solution and then manually inserted into the skin for 1 min before removal. Subsequently, a cotton swab was gently rubbed on the skin to collect any remaining calcein after MN insertion.

### 2.6. Fluorescence laser scanning microscopy

Delivery of the drug (calcein) through the porcine skin by porous-coated and non-coated MNs was visualized using fluorescence laser scanning microscopy (FLSM). Following drug delivery by individual MNs, porcine skin samples were cut into appropriate sizes and frozen in optimal cutting temperature compounds. The embedded frozen blocks were cut into 50 µm thick sections in horizontal directions using a microtome (RM2235, Leica, USA). The resulting skin sections were then visualized using FLSM.

### 2.7. Porosity characterization, imaging and graphing

Stereo and scanning electron microscopy was performed to inspect the porosity of the porous-coated MNs. SEM images of various porous-coated MNs at different magnifications were obtained to ensure consistent porosity on their periphery. Interconnectivity between the pores was also inspected. Pores from different porous-coated MNs were randomly chosen and visualized using the Image-J software package, and the diameters of various porous-coated MNs were measured to ensure uniformity of the porous-coated layer. Moreover, three-dimensional porosimetry analysis was performed to investigate the morphology of porous polymer-coating using Porosimeter (Company: micromeritics, Model: AutoPore IV 9520). Three samples with 5%, 10% and 15% porogen concentration in the polymer coating solution were prepared for these tests. The operating specifications were as follows: penetrometer parameters: sample weight: 0.4592 g, max. head pressure: 4.45 psia, pen. volume: 5.2835 ml. Hg parameters: adv. contact angle: 130°, rec. contact angle: 130°, Hg surface tension: 485 dynes cm^−1^, Hg density: 13.5335 g ml^−1^

### 2.8. Quantification of rhodamine B dye and lidocaine delivery

First, rhodamine B dye was used as a drug sample for the drug delivery test. This involved dissolving 1 g of rhodamine B in 20 g of DI water to make a 5% (w/w) solution. Porous-coated and non-coated MNs were dipped into the dye solution and subsequently inserted in an aqueous gelatin gel and PBS mixture. After the delivery of rhodamine B, lidocaine was then used as a sample drug. In this case, a 5% (w/w) solution of lidocaine was prepared in water. Lidocaine delivery was analysed using both non-coated and porous-coated MNs with PBS as the receiving medium. Each experiment was performed in triplicate, and mean values were used for the analysis. The concentration of the delivered drug (i.e. rhodamine B or lidocaine) in the receiving medium was measured by UV spectrometry, and a standard curve was obtained by derivatizing the known concentrations of rhodamine B and lidocaine. The concentration of the delivered drug was then plotted on a standard curve and the masses of the delivered drug via the porous-coated and non-coated MNs were calculated.

## 3. Results and discussion

### 3.1. Porous PLGA-coating process and porosity characterization

**Figure 3.**
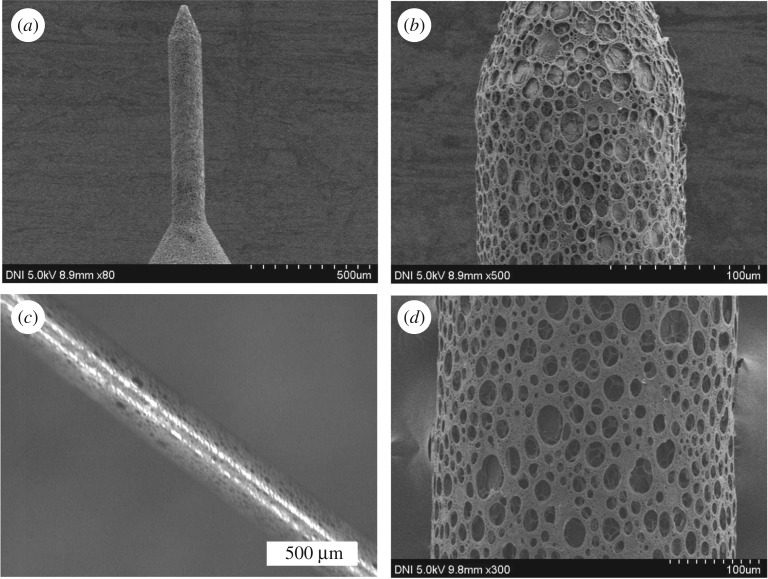
SEM and stereo micrographs of SS MNs coated with a 15% gelatin porous PLGA polymer. (*a*,*b*) SEM micrographs of a type A MN. (*c*) Stereo and (*d*) SEM images of a type B MN. The type A and B MN geometries possess lengths of 600 and 4000 µm, respectively, and diameters of 120 and 300 µm, respectively.

SS MNs were coated with a thin uniform layer of porous PLGA to provide a high delivery rate using a dipping method. PLGA, which is a biodegradable and biocompatible polymer, was used as the coating polymer and aqueous gelatin porogen was used to introduce pores into the polymer layer [31]. Both the ratio of aqueous gelatin to polymer solution and the homogenization rate affect the porosity of the porous layer. Note that the porogen ratio refers to the volume ratio of the porogen solution in the entire mixture. As reported previously, increasing the porogen ratio and decreasing the homogenization rate increases the pore size and vice versa [32]. Stereo microscopy and SEM were used to inspect the porosity and uniformity of the polymer coating on the MNs. Pores from different porous-coated MNs were measured, and the diameters of various porous-coated MNs were visualized by Image-J to ensure uniformity of the porous-coated layer (see electronic supplementary material, figure S4). All porous-coated MNs have essentially similar diameters within a narrow range (134–136 µm), demonstrating uniformity of the porous-coated layer. Figure 3 shows the stereo micrograph and SEM images of the porous-coated type A and B MNs at various magnifications. The bright field stereo micrograph reveals that all MNs were uniformly coated with porous PLGA. The SEM images further confirmed that the porous coating is a uniform thin layer, and that it does not alter the needle architecture. Additionally, the polymer layer is highly porous with uniformly distributed pores on the MN peripheries. The results indicate good interconnectivity between the pores, providing pathways on the MN peripheries for the passage of drug formulations (see electronic supplementary material, figure S3). We inspected the morphology of porous MNs with different porogen concentration by three-dimensional porosimetry analysis. Table 1 shows the results of the experiments. Results revealed that as the porogen concentration increased in the polymer coating solution, a significant increase in pore area, average diameter and porosity was observed.

**Table 1.** Three-dimensional porosimetry intrusion data summary.

sample	total intrusion volume	total pore area	average pore diameter	bulk density at 0.52 psia	apparent (skeletal) density	stem volume used (%)	porosity (%)
5% porogen	0.6817 ml g^−1^	41.188 m^2^ g^−1^	1.8955 µm	0.7327 g ml^−1^	1.4641 g ml^−1^	69	45
10% porogen	1.0648 ml g^−1^	63.414 m^2^ g^−1^	2.3381 µm	0.5336 g ml^−1^	1.1287 g ml^−1^	80	57
15% porogen	1.5245 ml g^−1^	73.623 m^2^ g^−1^	6.0791 µm	0.4149 g ml^−1^	1.1287 g ml^−1^	80	63

### 3.2. Effect of surface modification on interfacial adhesion

**Figure 4.**
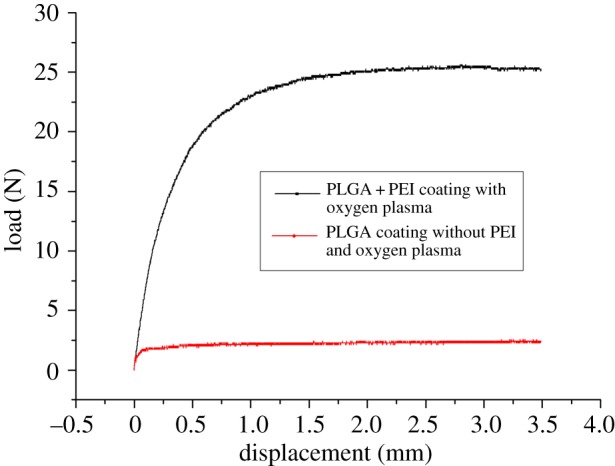
Results of the peel-off tests comparing the interfacial adhesion between the MN surface and the polymer layer with and without surface modification.

The results also revealed that the interfacial adhesion between the SS MNs and the polymer layer was relatively weak due to differences in their mechanical and physical properties. In order to obtain a stronger interfacial adhesion between these surfaces, the MN surfaces were modified. Without affecting the bulk properties of the MNs, the MN surfaces were first treated with oxygen plasma, which introduces carboxyl groups on the MN surfaces, and then subjected to a chemical reaction involving PEI (a polymer with repeating amine units) to improve the interfacial adhesion of the polymer coating. Following these surface modification steps, PLGA was coated onto the MNs. This approach was intended to significantly improve the coating stability between the metal surface and the porous polymer matrix. Peel-off tests with and without these surface modifications were performed to determine their effect on adhesive strength. These tests involved individually preparing the specimens and testing each of them in a UTM. The testing device applied a tensile force to the interface between the substrate and polymer coating, causing detachment of the coating. The results from these tensile tests indicate that the surface modified with oxygen plasma and PEI coating (i.e. the polymer-coated surface) has a higher adhesive strength than for the non-modified surface, as shown in figure 4. The average failure loads measured for the modified and non-modified surface coatings were 25 and 2.2 N, respectively. Without the surface modification, the polymer coating peels off the MN surface during insertion into the skin. By contrast, the polymer layer has sufficient adhesive strength to the metal surface to successfully penetrate into the skin when the MN surface is modified.

### 3.3. Skin penetration capability of porous polymer-coated microneedles

**Figure 5.**
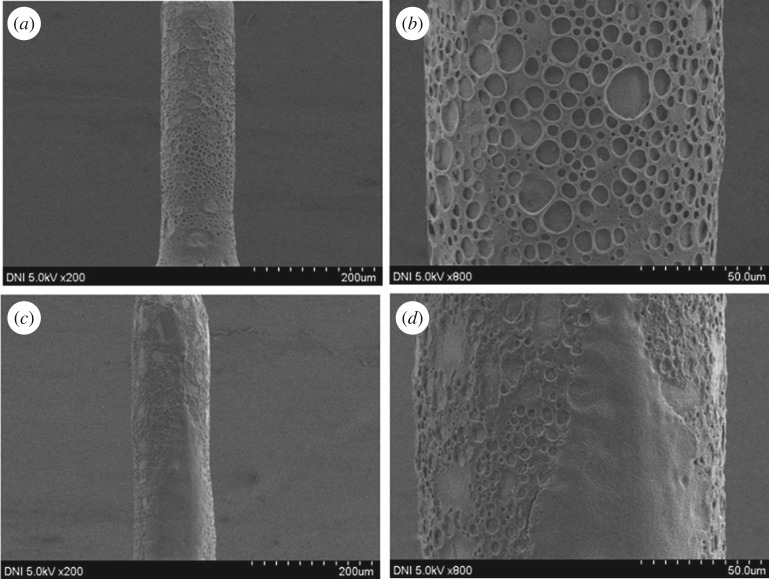
(*a*,*b*) SEM micrographs of porous PLGA-coated MNs with low and high magnifications before insertion in the porcine skin. (*c*,*d*) SEM micrographs of porous PLGA-coated MNs with low and high magnifications after the insertion in the porcine skin.

The skin penetration capability of MNs is essential for drug delivery as well as for sampling the interstitial fluid from inside the skin. A variety of factors influences the penetration efficiency of MNs such as the insertion force, insertion speed and age of the skin [33]. The penetration capability of porous PLGA-coated MNs was characterized using SEM images before and after insertion in porcine skin (figure 5*a,b* and *c,d*, respectively). As seen, the porous-coating morphology on the MNs remained unchanged following insertion. The porous polymer-coating neither detached from the MN surfaces nor became damaged after insertion in the porcine skin, which confirms that the porous polymer-coating has sufficient mechanical strength to penetrate the skin for drug delivery applications. As shown in figure 5*c,d*, some skin tissues were present on the pores after insertion.

### 3.4. *In vitro* drug delivery in porcine skin

**Figure 6.**
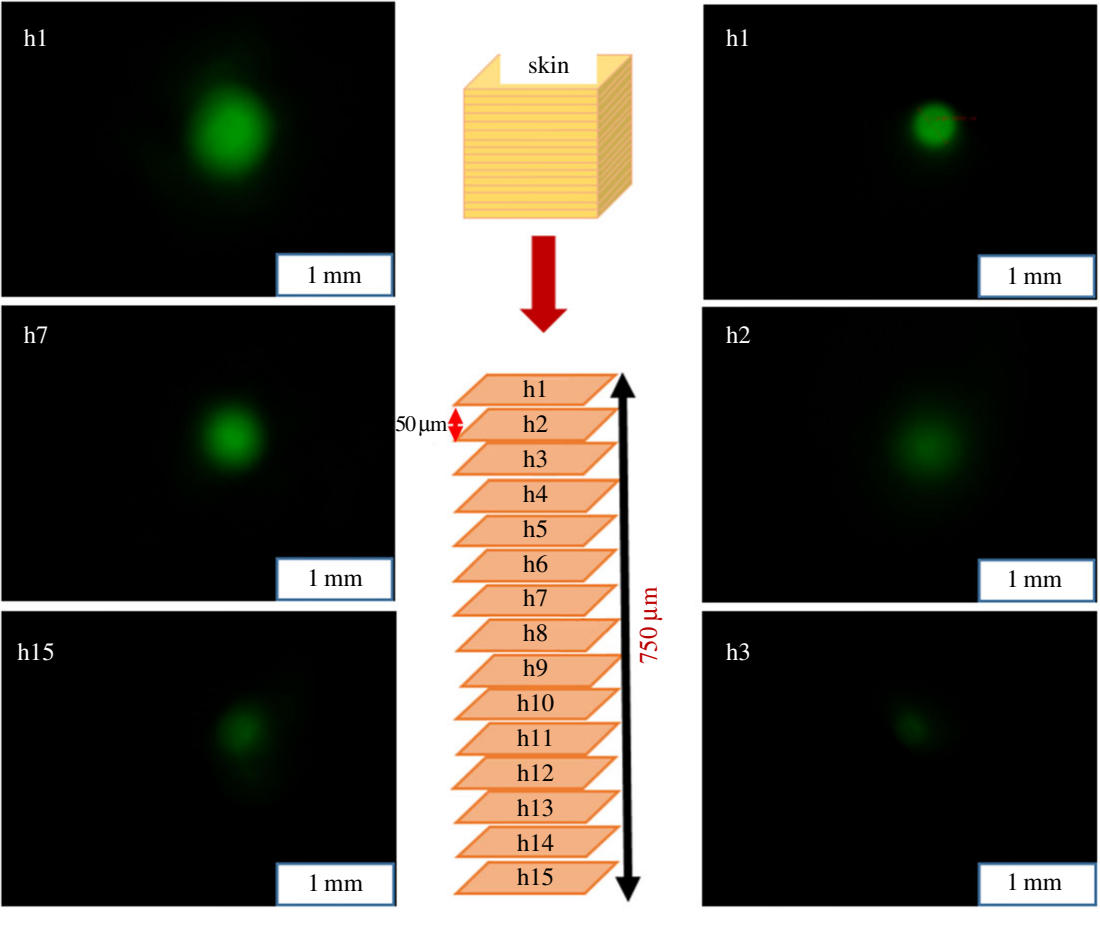
*In vitro* calcein delivery in porcine skin. The MNs were penetrated into the skin to a depth of 600 µm. The left side shows calcein delivery via porous polymer-coated MNs, whereas the right side shows delivery via non-coated MNs. h1 represents the top skin section, whereas h15 represents the deepest section for which calcein was detected. Calcein was delivered to depths of 750 and 100 µm by the porous-coated and non-coated MNs, respectively.

To investigate *in vitro* drug delivery by the porous polymer-coated and non-coated MNs in skin, porcine skin rather than live animals was selected for ethical reasons. Previous studies have shown that the delivery efficiency obtained *in vitro* in porcine skin is similar to that of *in vivo* in mice [34,35]. To this end, *in vitro* skin delivery of calcein by the porous-coated and non-coated MNs was performed in porcine skin and confirmed using imaging. The MNs were penetrated into the skin to a depth of 600 µm, the full length of MNs, which possessed a length of 600 µm and a diameter of 120 µm. The results show that calcein was successfully delivered into the skin to a depth of 750 µm by the porous-coated MNs. As shown in the left side of figure 6, h1 represents the top skin section of calcein delivery, whereas h15 represents the lowest skin section at which calcein was observed by FLSM. The thickness of each section is 50 µm.

By contrast, the calcein delivered in the skin by the non-coated MNs was found only to a depth of 100 µm when using a MN with identical dimensions to that used with the porous-coated MNs (i.e. length = 600 µm, diameter = 120 µm), as shown in the right side of figure 6. In this case, h1 represents the top skin section of calcein delivery, whereas h3 is the lowest skin section. Note that calcein was only observed on sections h1 and h2 and none was observed in the subsequent skin sections, each of which was 50 µm thick. These results confirm that the porous PLGA-coated MNs are capable of delivering a large amount of drugs to deeper skin sections.

### 3.5. Rhodamine B dye and lidocaine delivery

**Figure 7.**
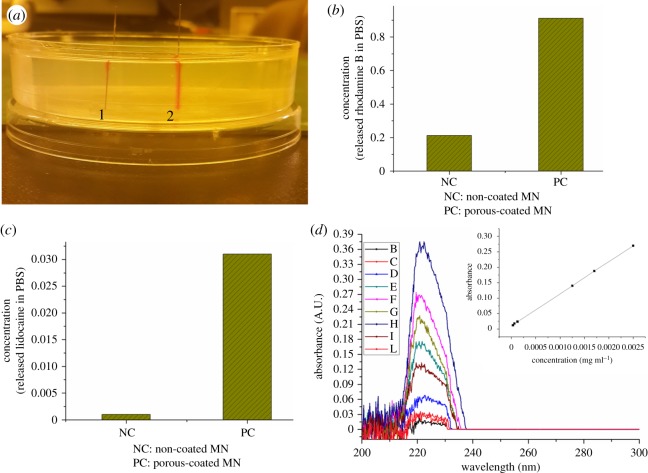
(*a*) Rhodamine B delivery in gelatin gel. 1: rhodamine B delivered by non-coated MNs; 2: dye delivered by porous polymer-coated MNs. (*b*) NC: rhodamine B delivered in PBS by non-coated MNs; PC: rhodamine B delivered in PBS by porous PLGA-coated MNs. (*c*) Lidocaine delivery in PBS. NC: delivery by non-coated MNs; PC: delivery by porous PLGA-coated MNs. (*d*) UV absorbance corresponding to known and drug delivered concentrations in the receiving medium. Inset is the standard curve with absorbance values and their corresponding drug concentrations.

In this study, rhodamine B dye delivery was examined in an aqueous gelatin gel and PBS using non-coated and porous-coated MNs. The transparency of this gel allowed real-time monitoring of dye delivery. As shown in figure 7*a*, the quantity of the sample delivered by the porous polymer-coated MNs exceeded that by the non-coated MNs, which demonstrates that most of the drug will remain on the skin surface when non-coated MNs are used. By contrast, relatively more drug can be delivered deeper into the skin when porous-coated MNs are used. A quantitative evaluation of this drug delivery was investigated using PBS as the receiving medium. The rhodamine B concentration in the medium after delivery with the porous-coated and non-coated MNs was measured using UV spectrometry, with the results given in figure 7*b*. As seen, relative to non-coated MN drug delivery, the porous-coated MNs promote a five times higher volume delivery when rhodamine B was used as the sample drug.

In the lidocaine delivery tests, porous-coated and non-coated MNs were dipped in a 5% (w/w) aqueous lidocaine solution and then immersed in PBS as the receiving medium. The lidocaine concentration in PBS was quantified by UV spectrometry. On the basis of the corresponding standard curve, the mass of the delivered lidocaine by an array of nine porous-coated and nine non-coated MNs was 2.06 and 0.08 µg ml^−1^, respectively. Clearly, the results indicate a 25 times increase in drug volume delivery for the porous-coated MNs relative to the non-coated MNs delivery when lidocaine was used as a sample drug, as shown in figure 7*c*. Figure 7*d* shows the UV spectrometry data (i.e. absorbance) to prepare the standard curve for drug delivery quantification. Overall, it is clear that the porous polymer coating method developed herein is very simple and can be used to deliver a variety of drugs with an increased delivery rate.

### 3.6. Effect of porosity on drug delivery rate

**Figure 8.**
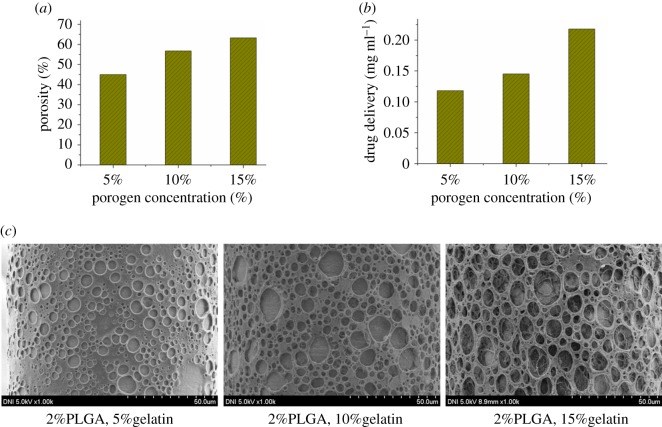
(*a*) Porosity of porous MNs with different porogen concentrations. (*b*) Drug delivery by porous MNs with different porogen concentrations. (*c*) SEM images of porous MNs with different porogen concentrations.

Figure 8 shows the effect of porogen concentration (aqueous gelatin) on the mass of drug delivered. The porosity of the porous polymer coating on the MNs was calculated with different porogen concentrations using three-dimensional porosimetry analysis. As the porogen concentration in the coating solution was increased from 5%, 10%, to 15% (w/w) of the total coating solution, a significant increase in the porosity of the polymer coating was observed (45%, 57%, to 63%, respectively) as shown in table 1 and figure 8*a*, while figure 8*c* shows the SEM images of the corresponding porous MNs with the porogen concentration of 5%, 10% and 15%. The amount of lidocaine delivered by the array of nine porous polymer-coated MNs with 5% (w/w) porogen was 1.03 µg ml^−1^. When the porogen concentration in the coating solution was increased to 10% and 15% (w/w), the delivery of lidocaine increased to 1.29 and 2.06 µg ml^−1^, respectively, as shown in figure 8*b*, which is more in amount in comparison with the results previously reported by Vrdoljak *et al*. [13]. Clearly, drug delivery increased concomitantly with porosity. This increase can be attributed to an increase in the sizes and number of pores in the polymer coating on the MNs, which was determined by the amount of porogen in the coating solution. Therefore, a desired quantity of drug can be delivered by adjusting the porous coating porosity on the MNs.

## 4. Conclusion

An easy coating method for SS MNs with a porous polymer (PLGA) layer was developed to deliver a variety of drugs with high delivery rates. The polymer layer was highly porous and uniform, and the pores were distributed evenly on the MN periphery. Moreover, the pores were highly interconnected and can thus provide micro-passages on the MN periphery, which suggests that drug dosage may be controlled by way of continuous delivery. Plasma treatment and PEI coating were found to significantly improve the interfacial adhesion of the polymer coating to the MN surface. The average failure load recorded for the modified surface coating was 25 N, whereas that for the non-modified surface was 2.2 N. Calcein dye was successfully delivered into porcine skin by the porous polymer-coated MNs, demonstrating that the prepared MNs can pierce the skin easily without deformation, which was further confirmed with skin penetration tests. Similarly, lidocaine and rhodamine B dye were delivered in PBS medium by porous polymer-coated and non-coated MNs. For rhodamine B, drug delivery by the porous-coated MNs was five times higher than that by the non-coated MNs, whereas 25 times more lidocaine was delivered by the porous-coated MNs compared with the non-coated MNs. In the future, the porous-coated MNs fabricated herein could be connected to a micropump to develop automatically controlled drug delivery systems.

## Supplementary Material

Supporting Information on “Porous polymer coatings on metal microneedles for enhanced drug delivery” for the detailed description of type A and type B microneedles (MNs), MNs array, morphology of porous coating on MNs tip and pores size.

## Data accessibility

Our supplementary data for the detailed description of type A and type B MNs, MNs array, morphology of porous coating on MN tip and pores size are available in the electronic supplementary material.
