# Supplementary material for: Porous polymer coatings on metal microneedles for enhanced drug delivery
Source: R Soc Open Sci. 2018 Apr 18;5(4):171609. doi: 10.1098/rsos.171609 (PMC5936903; doi:10.1098/rsos.171609)
Supplement: Supporting Information on “Porous polymer coatings on metal microneedles for enhanced drug delivery” for the detailed description of type A and type B microneedles (MNs), MNs array, morphology of porous coating on MNs tip and pores size. [file rsos171609supp1.docx]

Supporting Information on “Porous polymer coatings on metal microneedles for enhanced drug delivery” for the detailed description of type A and type B microneedles (MNs), MNs array, morphology of porous coating on MNs tip and pores size.

Fig. S1 shows the stereo micrograph of type A microneedle (MN). Type A MN was prepared by metal cutting. Section X of the MN shown in Fig. S1(A) was coated with porous polymer layer. Length of coated MN was 0.6mm whereas it had a dimeter of 0.12mm. Section X of MN was completely inserted in the porcine skin and in the PBS media for drug delivery tests. Fig.S1(B) shows the array of 9 MNs in the acrylic plate. Fig. S1(C) shows the complete geometry of the MN


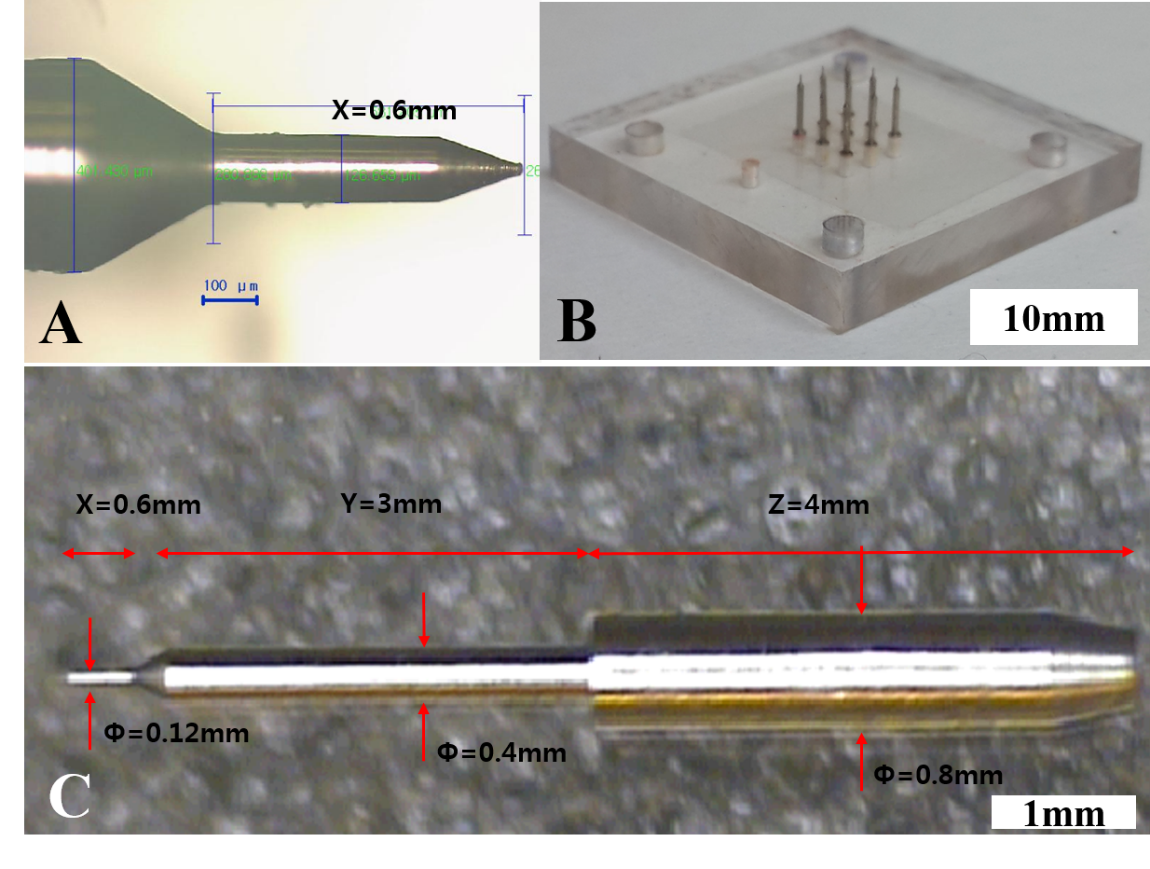


**Fig. S1.** Stereo micrograph of type. (A) microneedle. A. Magnified image of section X, (B) array of MNs in acrylic plate, (C) complete geometry of MN.

Fig. S2 shows the complete geometry of type B MN. 4mm long wire was cut from SS wire having a diameter of 0.3mm. Half of the MN was coated with porous polymer layer and some of the remaining un-coated part was inserted in the polydimethylsiloxane jig. Type B MN was only used for visual comparison of rhodamine B dye delivery in the gelatin gel shown in Fig.7(a). The transparency of this gel allowed real time monitoring of dye delivery.


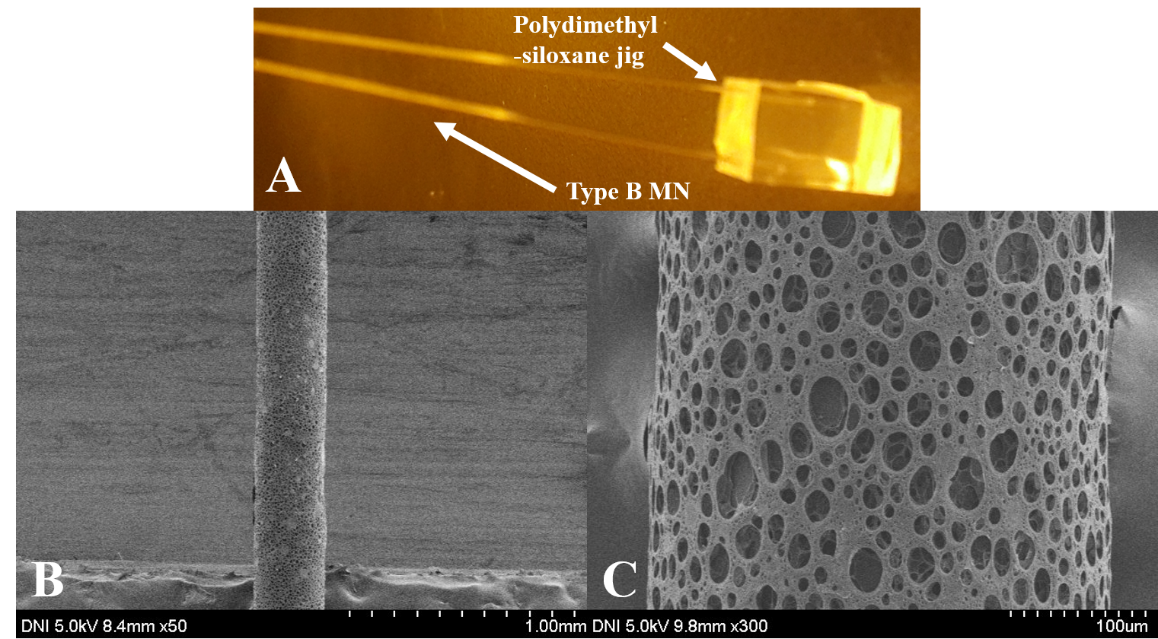


**Fig. S2.** (A) Stereo micrograph of type B microneedle, (B,C) SEM images with different magnifications.

Fig. S3 (A) shows the magnified image of type A MN. Clearly it can be seen in the figure that pores are uniformly distributed on MN and have good interconnectivity. (B) shows the effect of porous polymer coated layer on the tip of MN. It is clear from the figure that porous layer is also coated uniformly on the tip and pores are distributed on the tip as well.


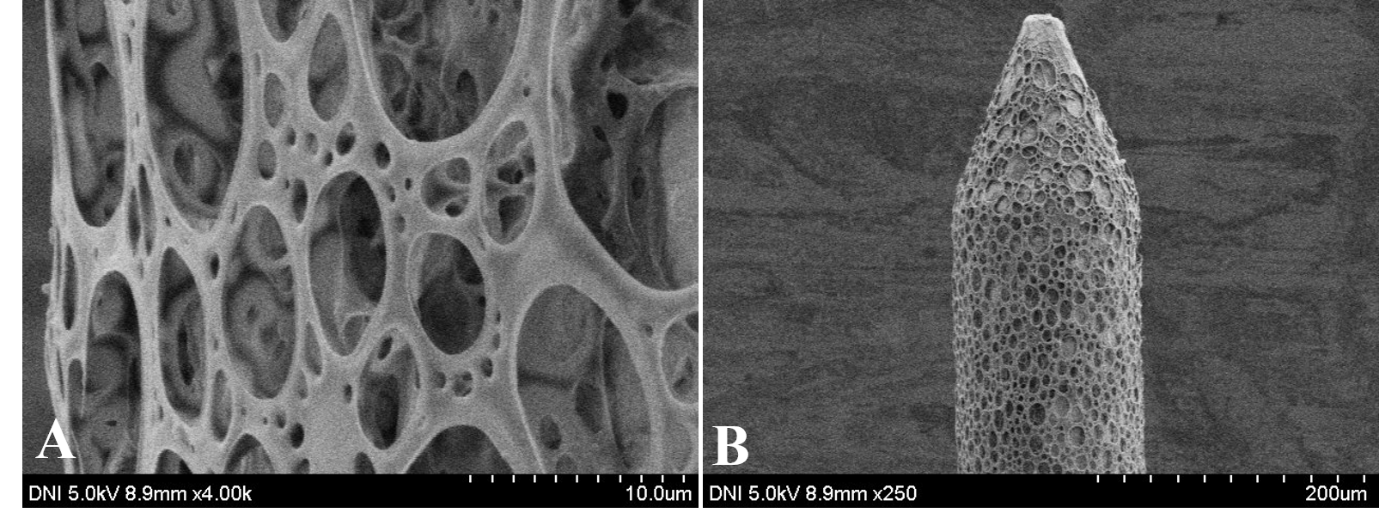


**Fig. S3.** (A) Magnified SEM of type A microneedle, (B) SEM image of the tip of type A microneedle.

Pores from different porous-coated microneedles were measured, and the diameters of various porous-coated microneedles were visualized by Image-J to ensure uniformity of the porous-coated layer. All porous-coated microneedles have essentially similar diameters within a narrow range (134–136 μm), demonstrating uniformity of the porous-coated layer. Porosity with different porogen concentration were also inspected. The data are shown in Fig. S4 along with pores size distribution graphs. The results revealed that as porogen concentration increased from 5%, 10% to 15% in the polymer coating solution, area of pores also increased from 41%, 51% to 66% respectively.


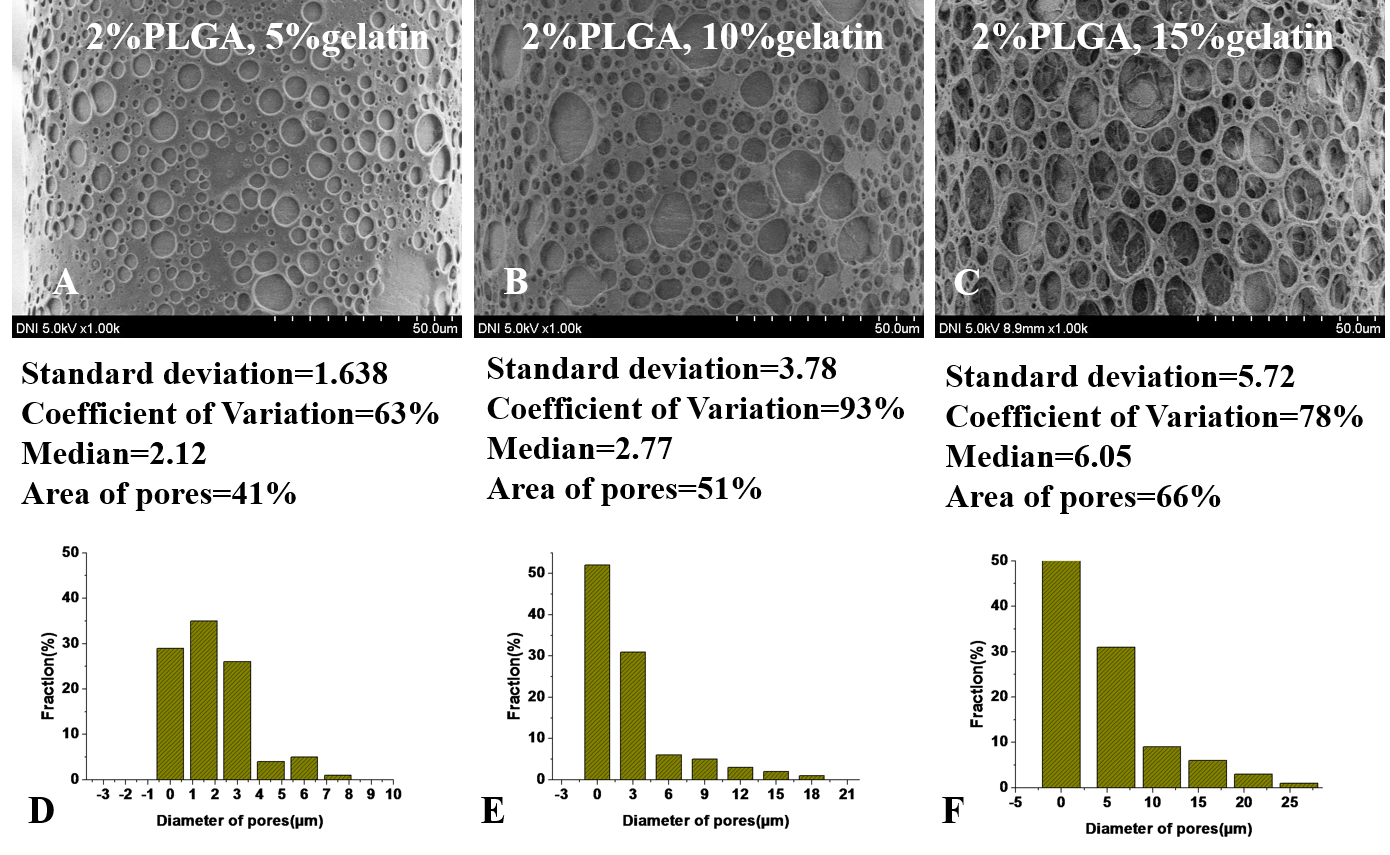


**Fig. S4.** Data of pores measured by Image-J software package. (A, B, C) SEM images of 5%, 10% and 15%porogen concentration porous coated MNs with 2%PLGA respectively. (D, E, F) are the corresponding pores distribution graphs.
